# Minnesota State Dental Society

**Published:** 1884-08

**Authors:** 


### EDITORIAL.

### Minnesota State Dental Society.

It was my good fortune to be present a tthe meeting of this Society in St. Paul on the 17th and 18th of July. This was the first annual meeting of this body, and from the way it performed its work, one might have supposed it had been in operation many years. Its membership is not inferior to that of most of the older societies, and it was a surprise to see so much interest and earnestness manifested by the members. They are taking hold of the subject of education and legal enactment for the protection of dental practice with a hearty good will; they have set in motion agencies for securing a law at the coming legislature, and from the energy manifested will doubtless succeed.

We were surprised to find two such growing, rushing, young cities as St. Paul and Minneapolis, so far to the northwest. They each contain about one hundred thousand inhabitants. Each one is the larger, when one is in it; but either one is large enough to satisfy any reasonable desire. They both present quite a metropolitan aspect, having almost all the advantages of larger cities. This has been an inviting field to the profession, as most of those who have been practicing here for any length of time, have attained a good degree of success. Some have gone a little into outside matters, and have been successful; indeed, one could scarcely live in St. Paul or Minneapolis without being carried into the current of speculation, and those who have kept within reasonable bounds have attained more or less of prosperity. We feel sure that from year to year we shall have good reports from the Minnesota Dental Society. Long may it live.

